# The Young Ravens of Physic

**Published:** 1896-08-15

**Authors:** 


### The Hospital

AN INSTITUTIONAL JOURNAL OF

The Medical Sciences and Hospital Administration,

WITH

AN EXTRA NURSING SUPPLEMENT.

VOL. XX.—No. 516.] [AUG. 15, 1896.

### THE YOUNG RAVENS OF PHYSIC.

THE believers in special providences often tell us that the young ravens of our rookeries and elsewhere are always plentifully supplied with the necessaries of life however numerous they may happen in any given season to be. Dr. Mitchell Banks, who, in his capacity of man of science, has his doubts about too many special providences, believes that young doctors, when they happen to be too numerous, are by no means sure even of a very moderate supply of the indispensable "wherewithal" to live. It has happened to Dr. Mitchell Banks to be appointed President of the Lancashire and Cheshire Branch of the British Medical Association; and he has, according to custom, faced his brethren in a presidential address. The subject was old, but perennially interesting. "The modern doctor can hardly live: why can't he?" That was the point of the address.

The Lancashire and Cheshire branch of the B.M.A. is one of the most important of all the branches of the Association. It is numerous, it labours in the midst of a strenuous industrial population, it feels the full pressure of the times, it is typical of most of the other great branches. What it feels they will feel, what it suffers they will not escape. Dr. Banks has attended within the past few years a great many meetings of the branch, and "during the past year," he tells his brethren, "he has been painfully struck by the often repeated mention of the very small fees which, in certain departments—notably that of midwifery—many members of our profession are forced to accept." The words "forced to accept" have an ominous sound, but unfortunately we cannot but believe they were used with full appreciation of their meaning. Dr. Banks, like a man of sense, does not consider that there is anything necessarily disgraceful in a low fee. On the contrary, he asserts with something like virtuous indignation that the "smallest fee, honourably earned from, and gratefully paid by, the poorest working man is a king's ransom in moral value compared with the great sums extorted by that prince of quacks, the fashionable London specialist." From which we may take it that the ways of some of our so-called "specialists" in the metropolis do not find very much favour with the honest and discriminating men upon whom falls the stern and actual labours of the most arduous of all the professions.

But why is the modern medical man in such piteous plight? Why is it, in Dr. Banks' energetic language, that notwithstanding "physical labours, mental fatigue, harassment and anxiety," unknown to any but ourselves, the "value of the fees" paid to us is so "utterly disproportionate" as to be hardly more than a mere acknowledgment of our services? "Can it be," he asks, "that the actual market value of our services is depreciating as science advances?" "That is not possible," he tells us; for "the article" we have to offer in the general market of the world "is of far better quality than it ever was as regards intrinsic worth." Perhaps it is the general "badness" of the times, he questions? Then it occurs to him that we may possibly be "falling lower and lower in the public estimation." All these are questions which medical politicians ought to ask themselves; and which Dr. Mitchell Banks did well to put into definite shape for his brethren. To be quite plain, the doctor is suffering tremendously at the present moment; and there must be reasons for it. What are the reasons? Our leaders in town and country are well advised in insisting upon a discovery of causes. If, when the causes have been convincingly brought to light, they can produce effectual remedies, the whole profession will give them its applauding gratitude.

Now, in a word, the cause of the depressed condition of medical practice is overcrowding. And, of course, the pessimist at once says that for overcrowding no remedy can ever be found. There we do not agree with him at all. For almost every ill under the sun a remedy can be found, provided the sufferer has adequate mental and moral capacity—not mental capacity only, but moral also. There are two remedies for the overcrowding of our profession. The one is to check the inflow, the other is organisation and mutual help. But the inflow of fresh members cannot be prevented. Population increases in all ranks, and all ranks seek work. A certain proportion of the middle class will force themselves into medicine, do as we may. And we have no right or even wish to forbid them. The only real remedy for the admitted evils of starvation wages is organisation and mutual help. But in ultimate analysis it is the factor of mutual help which is wanting. That is the reason why doctors starve. They have not the self-denial—the moral capacity, in short—to stand loyally by each other for mutual advantage. As a matter of fact, we do not believe that there are really too many doctors for the public needs. We may possibly admit that the consultants are too numerous; but certainly not the general practitioners. It is the latter who always complain; and it is they who cannot find in their hearts enough self-denial to enable them to bring about a better state of things. This is the word which experience dictates for the hour: "Let medical men combine; and let them stand loyally by each other in their combination." Then they will secure with the most absolute certainty a fair return for their services, exactly as all other organisations of men do who combine wisely and stand by each other honestly and loyally. It is individual selfishness which lies at the root of all the unprosperousness of the modern medical man.

